# Serum CA125 for Detection of Antibody-Mediated Rejection in Heart Transplantation Recipients

**DOI:** 10.3390/ijms27146499

**Published:** 2026-07-22

**Authors:** Irene González-Torrent, Lorena Pérez-Carrillo, Marta Delgado-Arija, Carlota Benedicto, Irene Carrasco-Hernández, Estefanía Tarazón, Esther Roselló-Lletí

**Affiliations:** 1 Clinical and Translational Research in Cardiology Unit, Health Research Institute Hospital La Fe (IIS La Fe), Avd. Fernando Abril Martorell 106, 46026 Valencia, Spain; irene_gonzalez@iislafe.es (I.G.-T.); lorena_perezc@iislafe.es (L.P.-C.); marta_delgado@externos.iislafe.es (M.D.-A.); carlota_benedicto@iislafe.es (C.B.); irene_carrasco@iislafe.es (I.C.-H.); 2 Doctoral Program in Biotechnology, Universitat Politècnica de València, Camino de Vera, s/n, 46022 Valencia, Spain; 3 Center for Biomedical Research Network on Cardiovascular Diseases (CIBERCV), Avd. Monforte de Lemos 3-5, 28029 Madrid, Spain

**Keywords:** carbohydrate antigen 125, antibody-mediated rejection, biomarker, heart transplantation

## Abstract

Although the potential role of carbohydrate antigen 125 (CA125) in acute cellular rejection (ACR) has been explored, its role in antibody-mediated rejection (AMR) remains unclear. Since CA125 is associated with inflammation and congestion, we hypothesized that CA125 could reflect key processes in AMR and complement the existing diagnostic methods. We analyzed 491 serum samples from heart transplant recipients undergoing first-year routine endomyocardial biopsies (EMBs). The results of EMB were classified as no-rejection, ACR, or AMR. CA125 levels were measured using a chemiluminescent immunoassay. CA125 concentrations were significantly higher in the AMR group (median 41 [IQR 26–78] U/mL) than in the no-rejection group (median 17 [IQR 11–27] U/mL; *p* < 0.01), regardless of post-transplantation time. In contrast, no significant differences were observed between the ACR and no-rejection groups (*p* = 0.124). Receiver operating characteristic analysis demonstrated strong diagnostic performance of CA125 for AMR (area under the curve = 0.821; *p* < 0.001). CA125 was an independent predictor of AMR (odds ratio, 5.04; *p* < 0.01). Our results demonstrated that elevated circulating CA125 levels are associated with AMR and exhibit promising diagnostic performance after heart transplantation, highlighting their potential as a non-invasive biomarker. This is further supported by the wide availability, standardization, and reproducibility of CA125 measurements in clinical practice.

## 1. Introduction

Heart transplantation (HT) remains the definitive therapeutic option for patients with end-stage heart failure (HF) in the absence of contraindications [1]. Nevertheless, acute rejection (AR) continues to be a major determinant of post-transplant prognosis. AR can manifest as acute cellular rejection (ACR) and/or antibody-mediated rejection (AMR) [2]. ACR is characterized by interstitial T-lymphocyte infiltration and myocyte necrosis [3], whereas AMR is characterized by endothelial cell activation, upregulation of cytokines, infiltration of macrophages, and microvascular thrombosis [4]. AMR, even without allograft dysfunction, is associated with a higher risk of cardiovascular mortality than ACR [5].

Endomyocardial biopsy (EMB) remains the gold standard for the diagnosis of AR. However, as an invasive procedure, EMB is associated with the risk of complications and is also subject to interobserver variability, which can cause under- or overtreatment [6]. Although various biomarkers for AR have been identified to reduce the need for repeated EMB, they still show several limitations that limit the scope for standardizing their use [2].

Carbohydrate antigen 125 (CA125), a complex glycoprotein, is a well-established tumor marker for ovarian cancer [7]. Beyond oncology, a growing body evidence supports the use of CA125 in the context of HF management, since serum levels of CA125 have been associated with disease severity and mortality [8]. The expression of CA125 is enhanced by inflammatory cytokines, and elevated circulating CA125 levels have been correlated with congestion and inflammation in HF [8].

Although CA125 has been explored in the context of ACR [9], its role in AMR remains unknown. Since AMR is associated with cytokine upregulation, microvascular dysfunction, and worse prognosis [4] and CA125 is linked to inflammatory and congestion-related pathways, we hypothesized that CA125 levels could reflect pathophysiological processes relevant to AMR and provide complementary information to established diagnostic methods.

## 2. Results

### 2.1. Clinical Characteristics

The clinical characteristics of the patients included in the study are summarized in Table 1. The median age was 55 [49–63] years: the majority of the patients were male (78%), and the most common indication for transplantation was non-ischemic dilated cardiomyopathy (43%). All groups were similar regarding variables such as age, body mass index, hypertension, diabetes, and dyslipidemia, among others. Nevertheless, in comparison with the no-rejection group, both ACR and AMR groups showed lower neutrophil and leukocyte counts and higher N-terminal pro-B-type natriuretic peptide (NT-proBNP) levels (*p* < 0.05). When rejection subtypes were compared, AMR showed further reductions in neutrophil and leukocyte counts in comparison with ACR (*p* < 0.05). In addition, the time between transplantation and obtaining the biopsy in ACR was significantly different from that in the no-rejection group (*p* < 0.001).

### 2.2. Serum CA125 Levels in Rejection

**Figure 1:**
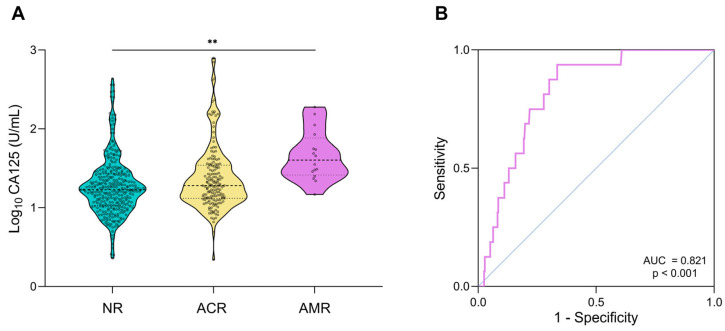
Serum CA125 levels for detecting cardiac allograft rejection. (**A**) Comparison of the log-transformed CA125 levels between the no-rejection, ACR, and AMR groups. Violin plots show both the median (dashed line) and the density of the data distribution in each group. ** *p* < 0.01. (**B**) Receiver operating characteristic (ROC) analysis of serum CA125 levels for detecting AMR. ACR, acute cellular rejection; AMR, antibody-mediated rejection; AUC, area under the curve; NR, no-rejection.

In the comparison between patients with ACR and those without (19 U/mL [13–35] vs. 17 U/mL [11–27]), no statistically significant differences in serum CA125 levels were observed (*p* = 0.124; Figure 1A). In contrast, serum CA125 levels were significantly higher in the AMR group (median, 41 U/mL [26–78]) than in the no-rejection group (median, 17 U/mL [11–27]; *p* < 0.01; Figure 1A). ROC curve analysis demonstrated the ability of CA125 to detect AMR, with an area under the curve (AUC) of 0.821 (0.744–0.898) and a standard error of 0.039 (*p* < 0.001; Figure 1B).

**Figure 2:**
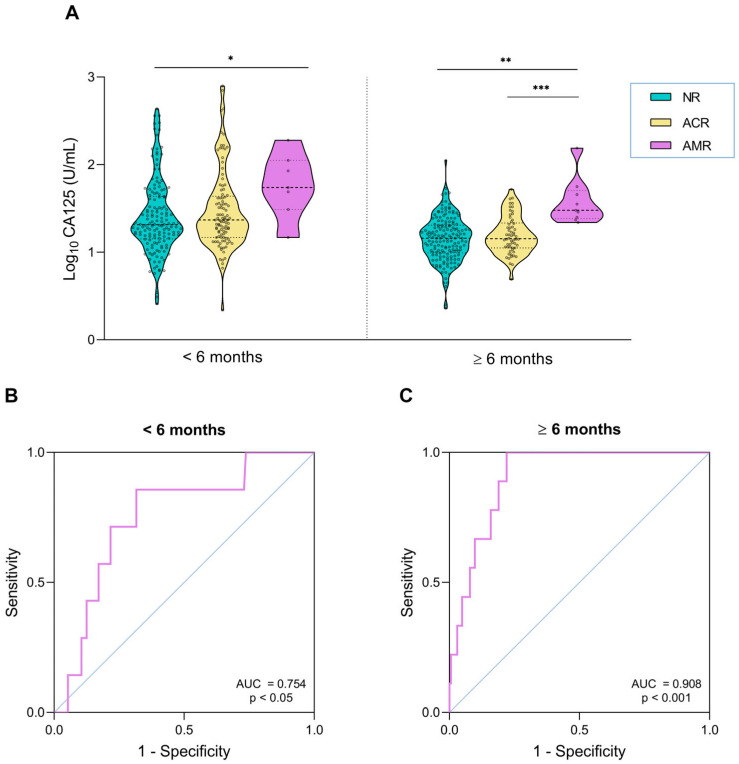
Serum CA125 levels for detecting cardiac allograft rejection at different post-transplant time points. (**A**) Comparison of the log-transformed CA125 levels between the no-rejection, ACR and AMR groups within the first 6 months and beyond 6 months post-transplant. Violin plots show both the median (dashed line) and the density of the data distribution in each group. * *p* < 0.05; ** *p* < 0.01; *** *p* < 0.001. (**B**,**C**) Receiver operating characteristic (ROC) analysis of serum CA125 levels for detecting AMR up to 6 months and after 6 months post-transplant. ACR, acute cellular rejection; AMR, antibody-mediated rejection; AUC, area under the curve; NR, no-rejection.

Next, we analyzed serum CA125 levels over time post-transplantation. Levels were stratified on the basis of the EMB surveillance time points, i.e., within the first 6 months and beyond 6 months post-transplant. As shown in Figure 2A, CA125 levels in ACR did not differ from those in the no-rejection group at any period, whereas CA125 levels in the AMR group were consistently higher than those in the no-rejection group in both periods. Within the first 6 months, the median level was 55 U/mL [31–112] in the AMR group versus 21 U/mL [14–43] in no-rejection (*p* < 0.05). Beyond 6 months post-transplant, the median level was 30 U/mL [24–51] in the AMR group versus 14 U/mL [10–20] in the no-rejection group (*p* < 0.01). Additionally, in the second period, CA125 levels were higher in the AMR group than in the ACR group (median 14 U/mL [11–22]; *p* < 0.001). ROC curve analysis confirmed the ability of CA125 to detect AMR in both periods, with an AUC of 0.754 (*p* < 0.05) for the first 6 months and an AUC of 0.908 (*p* < 0.001) for the period beyond 6 months (Figure 2B).

### 2.3. Diagnostic Evaluation of CA125 in AMR

The diagnostic performance of CA125 for detecting AMR with the standard cut-off value of 35 U/mL is summarized in Table 2. Over the 1-year follow-up period, CA125 showed a sensitivity, specificity, positive predictive value (PPV), and negative predictive value (NPV) of 56%, 84%, 15%, and 97%, respectively. Within the first 6 months post-transplant, the sensitivity, specificity, PPV, and NPV were 71%, 72%, 10%, and 98%, respectively. Beyond 6 months, the sensitivity, specificity, PPV, and NPV were 44%, 95%, 33%, and 97%, respectively.

Furthermore, to assess whether CA125 is an independent predictor of AMR, a multivariate regression model adjusted for age and sex was employed. Serum CA125 levels (>35 U/mL) were independently associated with an increased risk of AMR, with an odds ratio of 5.04 (95% confidence interval, 1.77–14.4; *p* < 0.01) and a C statistic of 0.727 (Table 3). To further evaluate whether this association was independent of graft dysfunction, left and right ventricular dysfunction was included as covariates in the multivariate model. After adjustment, elevated CA125 levels remained independently associated with AMR (*p* = 0.005).

Finally, since post-transplant CA125 levels may be influenced by pre-transplant CA125 concentrations, we incorporated baseline CA125 into the GEE model as a covariate to determine whether the association between post-transplant CA125 and AMR was independent of pre-transplant levels. CA125 measured at the time of biopsy remained independently associated with AMR (*p* = 0.028), whereas baseline CA125 was not significantly associated with AMR (*p* = 0.362). These results suggest that the observed association between CA125 and AMR is not explained by differences in pre-transplant CA125 levels.

## 3. Discussion

AR remains a major cause of compromised cardiac function and graft loss [10]. In the contemporary era, advancements in immunosuppressive therapy have contributed to reducing the incidence of ACR; however, the incidence of AMR has increased with the growing recognition of this condition [11]. The reported incidence of AMR varies widely from 1% to 40% because of the diversity of diagnostic criteria and institutional practices before the publication of the 2013 guidelines [10]. AMR represents a particularly challenging clinical entity, ranging from subclinical graft injury to catastrophic hemodynamic compromise [10]. Moreover, both symptomatic and asymptomatic AMR are associated with an increased incidence of cardiac allograft vasculopathy and cardiovascular mortality [12].

AR is currently diagnosed through routine surveillance EMBs, and the findings for ACR and AMR are evaluated and classified separately in accordance with established ISHLT criteria [12]. Since EMB is an invasive procedure and is subject to interobserver variability [13] several liquid biopsy-based methods have been recently implemented in clinical practice to reduce EMBs for the diagnosis of AR [12]. Distinguishing AMR from ACR can be challenging in certain cases. Accurate and prompt diagnosis of AMR is crucial, since it entails distinct management strategies, carries a higher risk of recurrence, and is associated with a worse prognosis than ACR [14]. Post-transplant monitoring of donor-specific antibodies (DSAs) is recommended in clinical practice guidelines, especially when AMR is suspected or confirmed by EMB [12]. However, AMR is widely known to exist in the absence of detectable levels of circulating DSAs [15], and detection of DSAs does not confirm the diagnosis of AMR [16]. Furthermore, several circulating molecules have been investigated as potential non-invasive biomarkers for AMR detection. Among these, natriuretic peptides have been associated with acute rejection; however, their diagnostic utility is limited by the lack of sufficient specificity to discriminate rejection from other complications after transplantation. Consequently, BNP and NT-proBNP are not recommended as stand-alone biomarkers for AMR diagnosis [17]. Inflammatory markers such as CRP and IL-6 could be potential indicators of early immune dysregulation, but current evidence remains inconsistent and their diagnostic value is unclear [17]. Beyond conventional inflammatory biomarkers, microRNAs have emerged as potential biomarkers for detecting AMR [14]. However their clinical implementation remains limited by the lack of consensus on the best miRNA candidates and assay protocols [17]. Therefore, the ideal screening test for AMR remains unknown.

CA125, which is widely used for ovarian cancer monitoring, has been recently explored in the context of HF management [18]. Several studies have demonstrated that serum CA125 levels are elevated in patients with HF and are associated with disease severity and poor prognosis [19]. Furthermore, Nuñez et al. reported that CA125-guided management of acute HF reduced rehospitalization rates in comparison with standard of care [20]. These observations suggest a potential role for CA125 in monitoring and guiding therapy in HF [18]. The biological role of CA125 is not fully understood, but cumulative evidence supports the positive association between CA125 levels and congestion and inflammation. The expression of CA125 by mesothelial cells is enhanced by inflammatory cytokines, such as tumor necrosis factor and interleukin-1, highlighting its potential involvement in the modulation of immune pathways in HF [8].

Although CA125 plays a well-established role in HF, its levels in patients with cardiac AR have been evaluated in only one study. López-Vilella et al. reported that this biomarker was not useful for predicting ACR; however, they hypothesized that in cases of very severe rejections accompanied by systemic congestion, CA125 levels could be elevated [9]. This is consistent with our results, since we did not observe significant differences in CA125 levels between patients with ACR and those without rejection. Despite these findings, the well-known association of CA125 levels with inflammation and congestion [8] indicated that it could be elevated in patients with AMR. In the present study focused on AMR, we observed elevated serum CA125 levels and strong capability to discriminate between patients with AMR and patients without rejection. Furthermore, this finding was observed in samples within the first 6 months and beyond 6 months post-transplant, indicating that CA125 had consistent discriminative capacity in both periods. Finally, multivariate analysis adjusted for age and sex demonstrated that CA125 was an independent predictor of AMR, reinforcing our finding. Moreover, the association between CA125 and AMR remained significant regardless of baseline CA125 levels, suggesting that the diagnostic ability of CA125 is independent of pre-transplant baseline concentrations.

CA125 offers several practical advantages over other biomarkers that facilitate its integration into routine clinical practice. Because of the routine use of CA125 measurements in oncology, these assays are widely available at any hospital laboratory. Moreover, CA125 is measured using standardized and highly reproducible methods, and its levels are not affected by processing time, sample type (serum or EDTA plasma), age, or renal dysfunction [8].

Our study has some limitations, and the results must be interpreted within this context. This investigation was conducted at a single center, necessitating validation through a larger multicenter study wherein the effects of different treatments could also be analyzed. Our results should be interpreted with caution given small number of AMR events. Nevertheless, we contend that our findings provide substantial evidence and represent a necessary step to support future research wherein these limitations can be addressed.

## 4. Materials and Methods

### 4.1. Study Design

This study was conducted using consecutive serum samples collected at the time of routine EMB from patients undergoing HT (age, >18 years). All consecutive patients who underwent primary heart transplantation at La Fe University and Polytechnic Hospital between July 2017 and December 2025 were considered eligible for inclusion. No additional clinical selection criteria were applied for study participation beyond those required for heart transplantation according to the institutional transplant program and current international guidelines. Patients who had received multiorgan transplantation or retransplantation or died during the initial postoperative period were excluded. All patients were maintained on a standard immunosuppressive regimen, consisting of a calcineurin inhibitor (predominantly tacrolimus), an antiproliferative agent (mycophenolate mofetil or mycophenolic acid), and corticosteroids. When clinically indicated, the immunosuppressive regimen was modified, including conversion to mTOR inhibitors.

AR episodes were evaluated in accordance with the International Society of Heart and Lung Transplantation (ISHLT) consensus report [3]. The histological and immunological findings in the EMBs were assessed by an expert pathologist blinded to clinical information. A second pathologist reviewed only cases with uncertain findings to reach a consensus.

**Figure 3:**
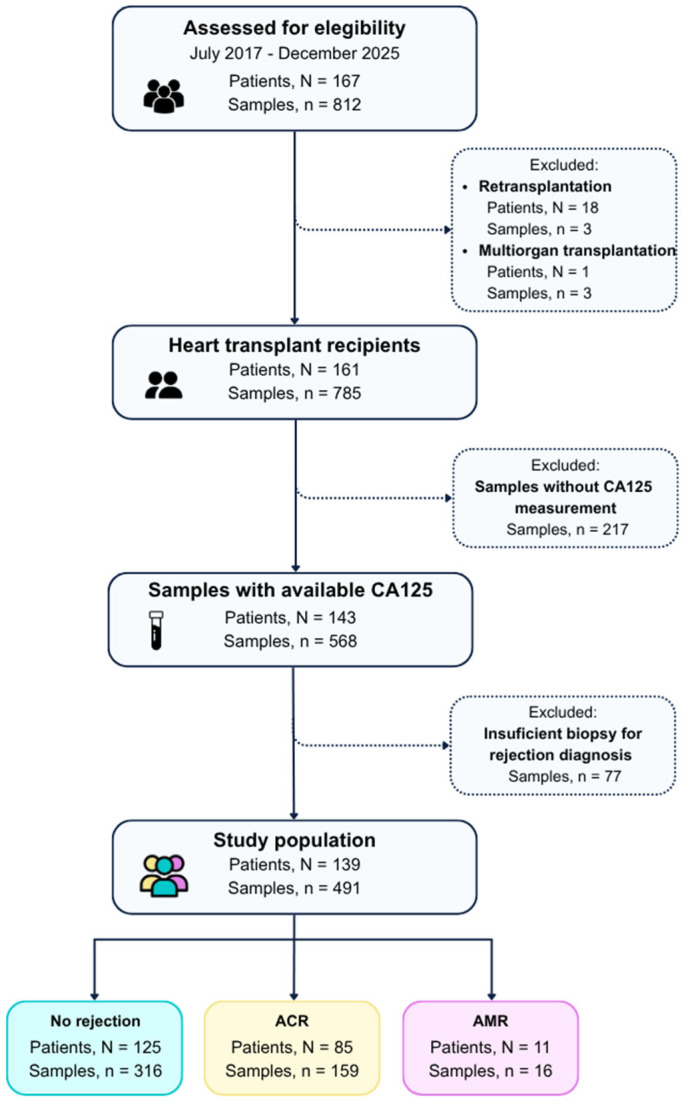
Samples and patients included in this study. Flow diagram showing patient and sample enrollment, eligibility and exclusions, as well as the allocation depending on the type of cardiac rejection for the final analysis. ACR, acute cellular rejection; AMR, antibody-mediated rejection. This study was approved by the Ethics Committee (Biomedical Investigation Ethics Committee of University and Polytechnic Hospital La Fe of Valencia, Spain). It was conducted in accordance with the principles outlined in the Declaration of Helsinki [21]. Before sample collection, informed consent was obtained from all patients.

The samples and their associated clinical data were collected during follow-up visits. This study was conducted using consecutive samples with available CA125 measurements and complete EMB diagnostic information obtained during the study period. A total of 491 paired serum samples and EMB evaluations from 139 patients were included. Patients could contribute more than one paired sample during follow-up, depending on the availability of concomitant CA125 measurements and EMB results. Nineteen patients (13.7%) contributed a single observation, whereas 120 patients (86.3%) contributed repeated observations ranging from two to seven follow-up visits per patient. Among the included samples, ACR was observed in 159 samples from 85 patients, AMR was detected in 16 samples from 11 patients, and 316 samples from 125 patients showed no evidence of AR (Figure 3).

### 4.2. Measurement of CA125 Levels

CA125 levels were measured as part of routine clinical assessment at our hospital during the post-HT follow-up by the Clinical Analysis Laboratory (Quality Certificate, ISO-9001:2015) using standardized protocols. Venous blood was collected by venipuncture and centrifuged immediately, and serum CA125 levels were quantified using the CA125 II chemiluminescent microparticle immunoassay on an Alinity i analyzer (Abbott, Green Oaks, IL, USA). The lower limit of detection was 1 U/mL.

### 4.3. Statistical Analysis

For continuous variables, data were presented as mean ± standard deviation or median and interquartile range, depending on normality assessed by the Shapiro–Wilk test. For categorical variables, data were presented as absolute and relative frequencies.

Given the longitudinal structure of the dataset and the variable number of repeated measurements available for each patient, generalized estimating equation (GEE) models were employed to account for the effects of repeated measures, with Patient ID serving as the subject variable to identify individual patients within the dataset. The correlation structure was assumed to be first-order autoregressive AR1. Continuous variables were compared using a GEE linear regression model with identity link, and categorical variables were compared using a GEE binary logistic model with a logit link. Both models included ACR or AMR as a fixed effect.

The diagnostic performance of CA125 levels for the presence of AMR was evaluated using a binary logistic GEE model with a logit link. AMR (binomial) was specified as the dependent variable and log-transformed CA125 levels as the fixed effect. The predicted values from this model were used to construct the receiver operating characteristic (ROC) curve. Diagnostic accuracy metrics were calculated using the well-established cut-off point of CA125 (>35 U/mL) [8].

Finally, to determine whether CA125 levels were an independent predictor of AMR, a multivariate GEE model including sex, age and ventricular dysfunction was employed. To assess whether the association between post-transplant CA125 levels and AMR was independent of pre-transplant CA125 concentrations, a second GEE model was fitted including baseline (pre-transplant) CA125 as an additional covariate. An incremental approach was used for the continuous variables (the calculated odds ratios are related to the increase in the indicated continuous variable by a given amount), and dichotomous risk factors were coded with an indicator variable of 1 for having the condition and 0 for its absence. Discrimination was assessed using the C statistic. CA125 levels were log-transformed as log10(X) for modeling and visualization. A *p*-value < 0.05 was considered to define a statistically significant difference. All statistical analyses were performed using SPSS (version 20.0), R (version R-4.3.1), and GraphPad Prism (version 8.0).

## 5. Conclusions

In conclusion, elevated circulating CA125 levels are associated with AMR and show promising diagnostic performance. As a well-established clinical biomarker, CA125 could be repurposed for heart transplant surveillance, and standardized methods and prior regulatory approval can facilitate its rapid translation into clinical practice. Further prospective studies in larger cohorts are needed to validate these findings.

## Figures and Tables

**Table 1:** Patient characteristics at the time of biopsy and blood sample extraction.

	No-Rejection N = 125 (316 Samples)	ACR N = 85 (159 Samples)	AMR N = 11 (16 Samples)
Age (years)	55 [49–63]	53 [47–63]	53 [45–61]
Male sex (%)	99 [79]	71 [84]	8 [73]
Indication for cardiac Tx			
ICM (%)	42 [33]	27 [32]	3 [27]
DCM (%)	55 [44]	38 [45]	4 [36]
Other (%)	28 [23]	20 [24]	4 [36]
NYHA classification			
II (%)	25 [21]	20 [25]	2 [18]
III (%)	60 [50]	42 [52]	6 [55]
IV (%)	34 [29]	19 [24]	3 [27]
Time from Tx to sample collection (months)	6.1 [3.4–9.8]	4.7 [3.1–8.0] ^a^	6.5 [3.1–9.4]
Primary graft dysfunction (%)	57 [46]	39 [46]	7 [64]
CMV infection (%)	1 [0.3]	1 [0.6]	0 [0]
Body mass index (kg/m^2^)	25 [21–28]	23 [21–27]	21 [20–24]
Hypertension (%)	71 [57]	48 [56]	5 [45]
Diabetes mellitus (%)	57 [46]	39 [46]	4 [36]
Dyslipidemia (%)	79 [63]	48 [56]	5 [45]
Neutrophils (thousands/mm^3^)	4.2 [2.7–5.4]	3.7 [2.5–5.0] ^a^	2.6 [1.4–3.8] ^b,c^
Leukocytes (thousands/mm^3^)	6.5 [4.7–8.3]	5.9 [4.3–7.6] ^a^	4.8 [3.2–7.1] ^b,c^
Lymphocytes (thousands/mm^3^)	1.4 [1.0–2.0]	1.4 [1.1–2.0]	1.3 [0.5–2.0]
NT-proBNP (pg/mL)	439 [248–953]	616 [329–1424] ^a^	2145 [635–6876] ^b^

ACR, acute cellular, rejection; AMR, antibody-mediated rejection; CMV, cytomegalovirus; DCM, idiopathic dilated cardiomyopathy; ICM, ischemic cardiomyopathy; NT-proBNP, N-terminal fragment of B-type natriuretic peptide; Tx, transplantation. ^a^ *p* < 0.05 between no-rejection and ACR, ^b^ *p* < 0.05 between no-rejection and AMR, and ^c^ *p* < 0.05 between ACR and AMR. Data are shown as median and interquartile range, or as absolute and relative frequencies.

**Table 2:** Performance of serum CA125 levels to diagnose AMR.

Period	AUC	95% CI	SS	SP	PPV	NPV
1-year follow-up	0.821	0.744–0.898	56.2%	84.2%	15.3%	97.4%
<6 months post-Tx	0.754	0.589–0.920	71.4%	72.4%	9.8%	98.2%
≥6 months post-Tx	0.908	0.849–0.967	44.4%	95.1%	33.3%	96.9%

Cut-off point: CA125 levels > 35 U/mL. AMR, antibody-mediated rejection; AUC, area under the curve; CI, confidence interval; NPV, negative predictive value; PPV, positive predictive value; SS, sensitivity; SP, specificity; Tx, transplantation.

**Table 3:** Multivariate GEE analysis for detecting AMR.

Variable	OR	95% CI	*p* Value	C Statistic
Sex	2.65	0.66–10.7	0.169	0.727
Age (years)	0.99	0.94–1.05	0.717	
CA125 levels (>35 U/mL)	5.04	1.77–14.4	0.003	

AMR, antibody-mediated rejection; CI, confidence interval; GEE, generalized estimating equation; OR, odds ratio.

## Data Availability

The datasets used and/or analyzed during the current study are available from the corresponding authors on reasonable request.

## Abbreviations

The following abbreviations are used in this manuscript:

AR	Acute rejection
ACR	Acute cellular rejection
AMR	Antibody-mediated rejection
AUC	Area under the curve
CA125	Carbohydrate antigen 125
CAV	Cardiac allograft vasculopathy
DSAs	Donor-specific antibodies
EMB	Endomyocardial biopsy
GEEs	Generalized estimating equations
HT	Heart transplantation
HF	Heart failure
ISHLT	International Society of Heart and Lung Transplantation
NPV	Negative predictive value
NT-proBNP	N-terminal pro-B-type natriuretic peptide
PPV	Positive predictive value
ROC	Receiver operating characteristic
